# Changes in Drivers’ Visual Performance during the Collision Avoidance Process as a Function of Different Field of Views at Intersections

**DOI:** 10.1371/journal.pone.0164101

**Published:** 2016-10-07

**Authors:** Xuedong Yan, Xinran Zhang, Yuting Zhang, Xiaomeng Li, Zhuo Yang

**Affiliations:** 1 MOE Key Laboratory for Urban Transportation Complex System Theory and Technology, School of Traffic and Transportation, Beijing Jiaotong University, Beijing 100044, PR China; 2 Department of Civil, Environmental, and Infrastructure Engineering, George Mason University, Fairfax, Virginia, United States of America; Beihang University, CHINA

## Abstract

The intersection field of view (IFOV) indicates an extent that the visual information can be observed by drivers. It has been found that further enhancing IFOV can significantly improve emergent collision avoidance performance at intersections, such as faster brake reaction time, smaller deceleration rate, and lower traffic crash involvement risk. However, it is not known how IFOV affects drivers’ eye movements, visual attention and the relationship between visual searching and traffic safety. In this study, a driving simulation experiment was conducted to uncover the changes in drivers’ visual performance during the collision avoidance process as a function of different field of views at an intersection by using an eye tracking system. The experimental results showed that drivers’ ability in identifying the potential hazard in terms of visual searching was significantly affected by different IFOV conditions. As the IFOVs increased, drivers had longer gaze duration (GD) and more number of gazes (NG) in the intersection surrounding areas and paid more visual attention to capture critical visual information on the emerging conflict vehicle, thus leading to a better collision avoidance performance and a lower crash risk. It was also found that female drivers had a better visual performance and a lower crash rate than male drivers. From the perspective of drivers’ visual performance, the results strengthened the evidence that further increasing intersection sight distance standards should be encouraged for enhancing traffic safety.

## Introduction

Road traffic crashes are consistently one of the top ten causes of death worldwide, among which intersection crashes lead to substantial severe injuries or fatalities [1]. The intersection crash rate can be effectively reduced through improving intersection sight distance (ISD) [2–4]. Sight distance is the length of the roadway ahead that should be visible to the driver [5]. In order to timely detect potential conflict vehicles and permit the drivers to anticipate and avoid potential collisions at an intersection, each quadrant of an intersection should contain a triangular area formed by sufficient ISD free of sight obstructions [5].

Essentially, the triangular areas free of obstructions at intersections are equivalent to drivers’ intersection field of view (IFOV), which represents drivers’ horizontal visibility at intersections. To reduce the ISD-related crash risk, it is critical to understand the effect of drivers’ IFOV on the collision avoidance performance, which depends on each individual driver’s judgment, capabilities, and response to conflict vehicles in the emergent situation. Yan et al. indicated that even under an assumption of valid ISD design compliant with the AASHTO design standards, further enhancing IFOV can significantly improve emergent collision avoidance performance at intersections, such as faster brake reaction time, smaller deceleration rate, and lower traffic crash involvement risk [6]. However, it is not clear how different IFOVs influence the drivers’ eye movement patterns and how the changes in drivers’ eye movements further impact the collision avoidance performance.

It was reported that about 90% of driving information is captured through the eyes [7]. Eye movements are considered as the behavioral interface between visual attention and information acquisition from the driving environments [8]. Visual attention while driving is used to direct information processing resources to potentially important visual event [9]. Evidence from previous research has been established that driving performance depends on visual attention [10, 11]. Persistent and accurate visual scanning of the intersection traffic environment is of great importance in understanding and determining drivers’ performance. On the contrary, the lack of visual attention to relevant driving events is one of the main factors in traffic crashes [12, 13]. Through investigating 2,258 traffic crashes, Treat et al. concluded that inadequate lookout and inattention were the two leading causes of the crashes [14]. A study of 723 crashes found that 37.8% of the accidents were due to drivers’ inattention or perceptual errors [15]. At intersections, a large number of crashes were due to failures to manage speed and maintain attention [16, 17], which may lead to failures to observe and appropriately judge the distance or speed of oncoming vehicles [18] and to see relevant traffic signs or signals [19] and cross traffic [20].

Numerous studies indicated that a driver’s useful FOV (UFOV) plays a significant role in driving. UFOV is defined as the visual area in which useful information can be acquired without eye and head movements (within one eye fixation) [21]. It is directly associated with driver’s ability of visual information acquisition [7, 22–24]. A consequence of reduced UFOV is the larger number of eye movements required to identify the location of a target [25]. With a constrained range of focus, drivers are less likely to perceive the objects around them and thus more likely to brake too late for collision avoidance [26]. Thus, better IFOV can help driver enlarge visibility at intersections in order to earlier and effectively search for the critical information, such as conflicting traffic. A recent simulator study has shown that further increasing IFOVs at unsignalized intersections can improve drivers’ emergent collision avoidance performance under an assumption of valid ISD design [6]. However, little research has been conducted to explore how IFOV affects drivers’ eye movements, visual attention and the relationship between visual searching and traffic safety, especially at non-signalized intersection.

The purpose of the study is to examine whether better IFOV conditions at non-signalized intersections can further improve drivers’ visual performance during the collision avoidance process and analyze the relationship between drivers’ eye movements and traffic safety. A simulator-based experiment with three different IFOV conditions was conducted to test drivers’ collision avoidance performance and identify the patterns in drivers’ scanning activities such as gaze duration, number of gazes and average gaze duration by the eye tracking system. The experimental results of this study would lead to a better understanding of the relationships among driving performance, eye moments and traffic safety under different IFOV conditions and provide a reference for sight distance design at non-signalized intersections.

## Method

### Ethics statement

The research involving human participants in this study has been approved by the Beijing Jiaotong University's research committee (per IRB). The written informed consent form for the experiment was also signed by each participant in this study.

### Participants

The experiment was a 3 (IFOV conditions) × 2 (gender) mixed design with repeated measures on the factor of IFOV. Twenty-three participants (11 men and 12 women) were recruited from the local community. All of the participants held a valid driver’s license and had at least three years of driving experience. Those with simulator sickness problems that could affect their driving performance were excluded. Their ages ranged from 30 to 40, with an average age of 35 years and a standard deviation (S.D.) of 2.99 years. It should be noted that examining the age effect on drivers’ eye movements was beyond the scope of this study. To be eligible for inclusion, participants had to hold a valid driver’s license, have at least three years of driving experience and drive more than 20,000 kilometers per year. Those with health problems that could affect driving behavior were excluded. The experiment lasted for about 30 minutes for each participant, who was compensated with RMB500 (approximately US$80).

### Equipment

**Fig 1:**
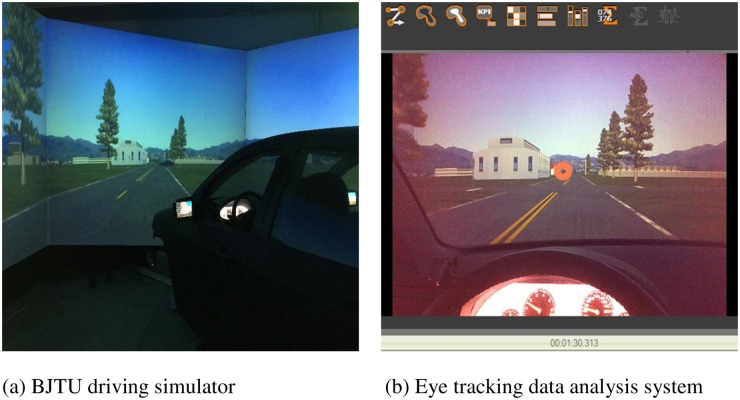
The BJTU driving simulator with eye tracking system. (a) BJTU driving simulator. (b) Eye tracking data analysis system.

The Beijing Jiaotong University (BJTU) driving simulator equipped with eye tracking glasses (ETG) was used to conduct the experiment and collect the datasets, as shown in Fig 1. The individual in this manuscript has given written informed consent to publish these case details. The BJTU simulator is a high-performance, high-fidelity driving simulator with a linear motion base capable of operating with one degree of freedom. It comprises a full-size vehicle cabin (Ford Focus) with a real operational interface, environmental noise and shaking simulation system, digital video replay system and vehicle dynamic simulation system. The simulated environment is projected at 300 degrees of a frontal/peripheral field of view at a resolution of 1400 × 1050 pixels and left, middle and right rear view mirrors. The software in the simulator lab allows for driving scenario design, virtual traffic environment simulation, and virtual road modeling.

Tracking eye movement has been considered as an appropriate way to measure drivers’ visual activities [13, 27–29]. In this experiment, ETG was designed as highly integrated glasses with a natural appearance to wear comfortably and used to collect participants’ binocular eye movements without restrictions of cab environment and the range of head motion. The ETG contains three video cameras: one is used to record the frontal views of participants and the other two are used to capture participants’ eye movements. The positions of participants’ fixation points and the tracks of their scanning activities distributed in the driving scenes are recorded in the video files. The specific information on the eye-movements variables (e.g., fixation, saccade and blink) such as time, duration and counts are all recorded in the eye tracker system.

### Scenario design

The scenarios of non-control intersections were selected to test the influence of IFOV on drivers’ crash avoidance performance and eye movements. From MUTCD (2009), the non-control intersections may exist when the average traffic volume from all approaches is lower than 2,000 units per day, where the approaching drivers should be able to see potentially conflicting vehicles in sufficient time to stop before reaching the intersection. According to AASHTO’s (2011), from the field observations at non-control intersections, drivers typically reduce their speed to 50% of their mid-block operation speed when approaching a non-control intersection. However, the traffic behavior patterns at non-signalized intersections in developing countries are different from the developed countries, where the common rules of “giving way” and “priorities” are not fully respected in most cases. Traffic conflicts at the intersections often occur because of drivers trying to “cut the corners” on the minor road. Additionally, the drivers on the minor are more likely to overspeed while approaching the intersections [30]. In China, the non-control intersections are typically seen in rural areas owing to lower traffic volume, where serious traffic collisions frequently happen especially when the intersection sight distance is restricted or drivers are speeding at the intersections.

The driving scenario designed in this study was the same as the study by Yan et al [6]. In this experiment, typical two-way two-lane non-control intersections with a 3.5 m lane width were created, and the speed limit was set at 80 km/h on the intersection’s major road and 60 km/h on the intersecting minor road. According to AASHTO’s ISD recommendations for non-control intersections [5], the lengths of clear sight triangle legs should be 75 m for a design speed of 80 km/h and 55 m for a design speed of 60 km/h. For the non-control intersections in this study, three IFOV conditions were designed based on the AASHTO’s standards. For the IFOV1 condition, the lengths of clear sight triangle legs are 80 m on the major road and 70 m on the minor road, which marginally satisfies the basic ISD requirement for non-control intersection. Based on the IFOV1 condition, the intersection angles of IFOV2 and IFOV3 conditions are increased by 5° successively. These different IFOV conditions are realized by moving the location of a building (sight obstruction) further from the corner of the intersection along the major road. Thus, the drivers when approaching the intersection from the major road can gradually have a wider horizontal view as the increase of the IFOVs. The design purpose of these three IFOV conditions is to explore whether better IFOV condition could further improve drivers’ visual performance during the crash avoidance process at non-signalized intersections, even though the sight distance has met the current intersection design standards.

A time-to-collision (TTC) sensor was used to realize the emergent scenario of pre-crash between the simulator vehicle on the major road and a conflict vehicle (from the right side of the major road) on the minor road that are simultaneously approaching the intersection, and the TTC threshold (the approaching time of the conflict vehicle to the conflict point at the intersection) was designed as 5 s in this experiment. When the TTC sensor was triggered by the simulator vehicle, the conflict vehicle would start to approach the conflict point at a constant speed of 72 km/h. The distance from the conflict vehicle’s initial position to the conflict point was set at 100 m upstream of the minor road. In such a situation, if drivers did not take any collision avoidance maneuvers, they would collide with the conflict vehicle.

### Experimental procedure

Upon arrival at BJTU, the features of driving simulator and ETG system were introduced to the participants, who were also indicated that the purpose of the experiment is to test the simulation fidelity of the BJTU driving simulator and they should adhere to traffic laws and drive as normal as what they do in reality. Before actual recording of drivers’ eye movements, the ETG system had to be calibrated for each driver to ensure an accurate collection of the fixation points. Then, a practice drive for at least 10 min was conducted for each participant to familiarize with the operation of the driving simulator. During the practice test, participants were advised to adhere to traffic laws and try different basic driving maneuvers such as acceleration, deceleration, braking and right/left turns. They were also notified that if they felt motion sickness or any other kind of discomfort, they were free to quit the experiment at any time. After a five-minute break, participants needed to perform three sets of formal experiments under different IFOV conditions in a random sequence to eliminate the experiment order effect. Each experiment included the same rural road network that composed of a series of typical two-way two-lane intersections. Among these typical intersections, only one intersection was randomly assigned to test drivers’ eye movements and driving performance. An emergent pre-crash scenario is a small probability event in real life, and drivers are unlikely to encounter one emergent conflict after another in short time. Hence, at least 10 minutes of normal driving in a typical rural road were inserted between each two sets of experiments to prevent from speculating about the experiment’s purpose and to minimize speculation to the repeated collision avoidance tests.

### Experimental data manipulation and dependent variables

During the experiments, raw data on driving behavior were sampled at 60 Hz while the datasets of eye movements were sampled at 30 Hz. Thus, it was necessary to synchronize the two types of datasets according to the time when the simulator arriving at the same location coordinates both in the SMI BeGaze video and in the driving behavior datasets, and then we recorded two time points and made a subtraction of two time points to match two datasets at the same time point when the simulator arrived at the same location coordinates in SMI BeGaze video and driving scenes.

**Fig 2:**
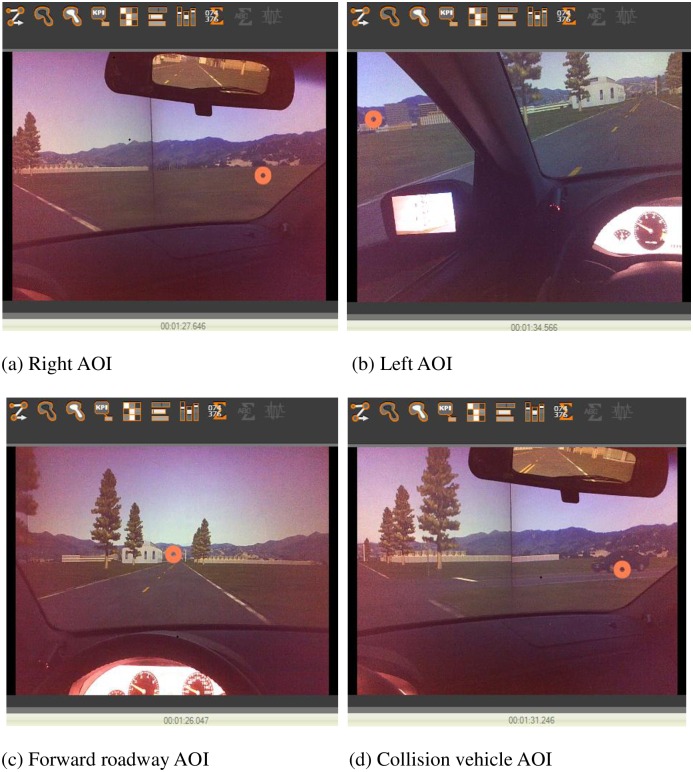
Classification of AOIs. a) Right AOI. (b) Left AOI. (c) Forward roadway AOI. (d) Collision vehicle AOI.

Based on the performance of 23 participants, 69 records were obtained from the three rounds of experiments under three IFOV conditions. Drivers’ scan paths (spatial sequence of fixations) and vehicle-operation performance variables were both recorded during the 5 s time period during which the conflict vehicle was triggered and traveled to the conflict point. The visual performance variables indicating eye movements include gaze duration (GD), number of gazes (NG), and average gaze duration (AGD) on the particular area of interest (AOI). The AOIs were divided into right area, left area, forward roadway and the conflict vehicle in this paper, as shown in Fig 2. Besides, vehicle-operation performance was indicated by brake time to conflict point (BTC) and collision or not (CON). Because three problem data were removed (the three eye movement data were failed to be correctly collected owing to drivers improperly adjusted the eye tracking glasses during the process of experiment), finally sixty-six records were used for statistical analyses. The hypothesis testing in this study was based on a 0.05 significance level. Specifically, the dependent variables are defined and explained as follows:

- Gaze duration (GD): Gaze duration was a cumulative duration in which a series of consecutive fixations fell within a particular AOI, and typically include several fixations and the relatively small amount of saccades between these fixations. The end time of the gaze was recorded when a fixation occurring outside of the AOI. GD was used to compare visual attention distributed among different AOIs. Especially, the gaze duration on the conflict vehicle (GDCV) was collected to analyze the effects of IFOV on drivers’ attention on the critical visual information.
- Number of gazes (NG): It was the number of gazes on the particular AOI, which can reflect drivers’ scanning activities and frequencies of visual search. Particularly, NGCV means the number of gazes on the conflict vehicle.
- Average gaze duration (AGD): It was calculated by dividing gaze duration by the number of gazes and reflects drivers’ visual search speed. A longer average gaze duration indicates slower visual search speed. Specifically, AGDCV was used to represent the average gaze duration on the conflict vehicle.
- Brake time to conflict point (BTC): BTC was measured from the time that the driver started a brake action to the time that he or she would arrive at the conflict point at the speed when braking. It was used to reflect drivers’ reaction and ability to decelerate in time to avoid a collision.
- Collision or not (CON): CON represented whether the driver collided with the conflict vehicle or not. It was an index for traffic safety evaluation.

### Statistical analysis method

Beyond the descriptive statistical analyses of the dependent variables, the logistic regression analysis was applied to test the effects of IFOV and gender on CON since it is a binary variable (collision or not), which can directly indicate the relationship between intersection field of view and driver’s collision risk under emergent situation. Additionally, the ANOVA method was used to investigate differences in the other continuous variables, such as GD, NG, AGD, and BTC, between the factors. The hypothesis testing in the following analyses was based on a 0.05 significance level.

## Experimental Results

### Collision rates (COR)

Table 1 shows the COR results of drivers’ collision avoidance and the logistic regression results for COR. The results showed that the COR results was significantly affected by different IFOV conditions (p = 0.044). There was a clear decreasing trend of COR as the IFOV condition increased. Respectively, the COR was 56.52% for IFOV1, 30.00% for IFOV2 and 21.74% for IFOV3. Compared with the condition of IFOV1, the COR under conditions of IFOV2 and IFOV3 was decreased by 67.7% and 79.8% respectively. Although the COR was not significantly affected by gender (p = 0.150), it was found that male drivers had a higher COR than female drivers (45.16% vs. 28.57%).

**Table 1:** Descriptive statistical results and logistic regression results for COR.

**Factor**		**COR**				**Total**
		**Collision**		**Non-collision**		
		**Count**	**Percentage**	**Count**	**Percentage**	
IFOV	IFOV1	13	56.52%	10	43.48%	23
	IFOV2	6	30.00%	14	70.00%	20
	IFOV3	5	21.74%	18	78.26%	23
Gender	Female	10	28.57%	25	71.43%	35
	Male	14	45.16%	17	54.84%	31
Total		24	36.36%	42	63.64%	66
**Logistic regression results for COR**						
**Factor**	B	S.E.	Wald	df	Sig.	Exp(B)
IFOV	---	---	6.269	2	0.044	---
IFOV2Vs. IFOV1	-1.129	0.657	2.952	1	0.086	0.323
IFOV3Vs. IFOV1	-1.599	0.672	5.652	1	0.017	0.202
Male Vs. Female	0.795	0.552	2.074	1	0.150	2.215
Constant	-1.016	.400	6.447	1	0.011	0.362

### Brake time to conflict point (BTC)

Different collision avoidance maneuvers (deceleration, acceleration or no avoidance maneuvers) were taken by the drivers to avoid colliding with a conflict vehicle when approaching a non-signalized intersection. The results suggested that the majority of drivers considered deceleration avoidance maneuver to the safest option for avoiding a collision with a conflict vehicle when crossing an intersection (77.27% of the participants), whereas only 12.12% of them chose acceleration collision avoidance and 10.61% of them took no collision avoidance. Thus, the influences of IFOV conditions and genders on the deceleration avoidance maneuver indicated by BTC were analyzed in this study.

**Fig 3:**
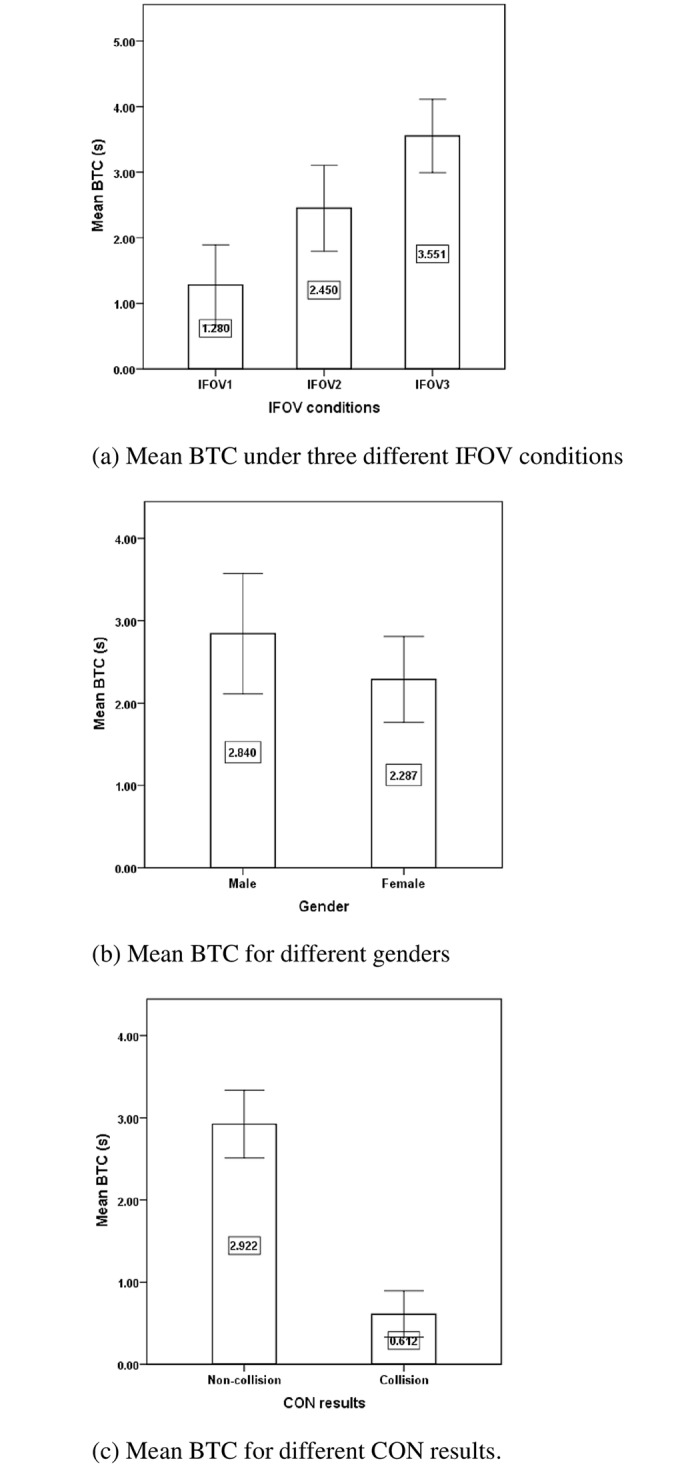
Mean BTC for different factors and CON results. (a) Mean BTC under three different IFOV conditions. (b) Mean BTC for different genders. (c) Mean BTC for different CON results.

Table 2 shows the descriptive statistics and ANOVA results for the BTC during the process of collision avoidance with the conflict vehicle. It was found that the IFOV conditions (F = 17.33, p < 0.001) significantly influenced the BTC, while gender had a marginally significant effect on the BTC (F = 3.044, p = 0.088). The BTC was lowest under IFOV1 condition (M = 1.280 s, S.D. = 1.148 s), followed by the conditions of IFOV2 (M = 2.450 s, S.D. = 1.182 s) and IFOV3 (M = 3.551 s, S.D. = 1.194 s). Fig 3-a clearly showed that the mean BTC increased as drivers’ IFOV conditions improved, which implied that the drivers took collision avoidance maneuvers earlier with increasing IFOV. Fig 3-b showed the mean BTC for males and females. Male drivers had longer mean BTC than female drivers (M = 2.840 s, S.D. = 1.607 s vs. M = 2.287 s, S.D. = 1.401 s), which implied male drivers braked earlier than female drivers. In addition, the BTC significantly influenced the CON results (F = 26.649, p<0.001), and drivers had longer BTC (M = 2.922 s, S.D. = 1.322 s) in non-collision group than the collision group (M = 0.612 s, S.D. = 0.367 s), as shown in Fig 3-c. The result implied that drivers were less likely to have a collision if they took deceleration collision avoidance maneuvers earlier.

**Table 2:** Descriptive statistics and ANOVA results for BTC.

Variable		BTC (s)					F-ratio	P-value
		Count	Mean	S.D. ^a^	Min	Max		
IFOV	IFOV1	16	1.280	1.148	0.050	4.350	17.33	0.000
	IFOV2	15	2.450	1.182	0.417	4.233		
	IFOV3	20	3.551	1.194	0.845	5.183		
Gender	Male	21	2.840	1.607	0.050	5.183	3.044	0.088
	Female	30	2.287	1.401	0.183	4.712		
Total		51	2.515	1.499	0.050	5.183		

^a^ S.D. = standard deviation.

### Gaze duration (GD)

**Fig 4:**
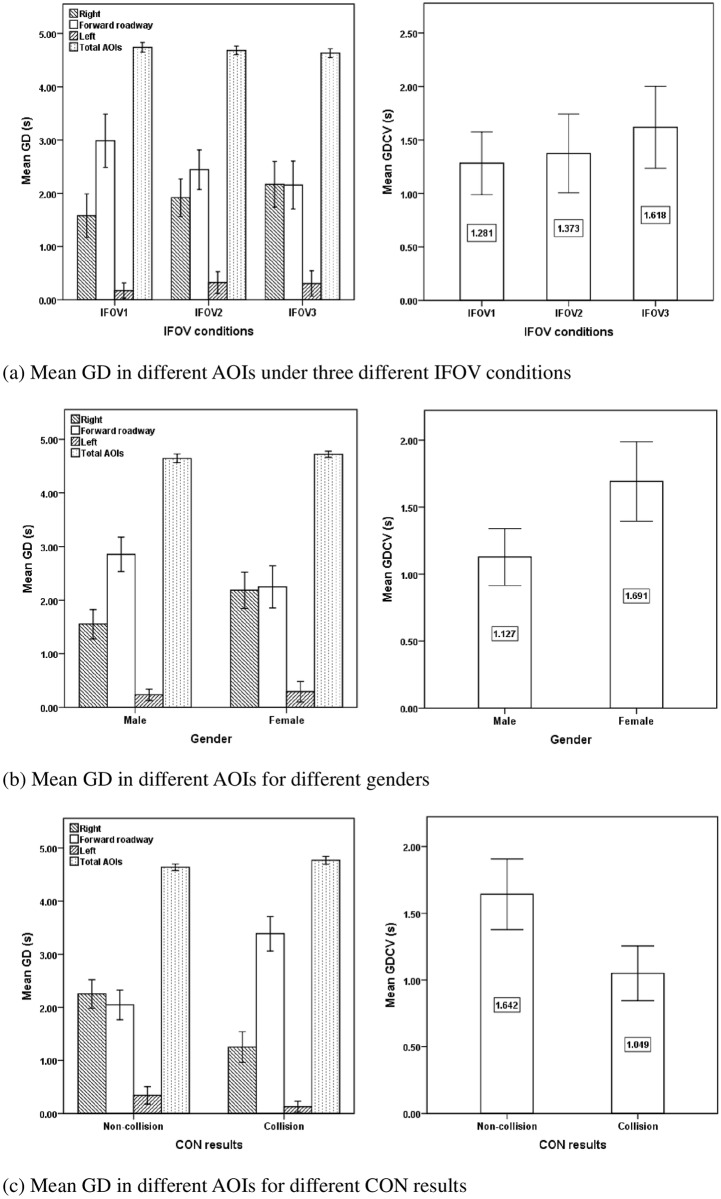
Mean GD in different AOIs for different factors and CON results. (a) Mean GD in different AOIs under three different IFOV conditions. (b) Mean GD in different AOIs for different genders. (c) Mean GD in different AOIs for different CON results.

Table 3 shows the descriptive statistics and ANOVA results for the gaze duration in different AOIs during the process of collision avoidance. The IFOV conditions significantly influenced the gaze duration in the forward roadway AOI (F = 4.130, p = 0.021) and had a marginally significant effect on the right AOI (F = 2.647, p = 0.079), while there was no significant effect on left AOI (F = 0.93, p > 0.1), total AOIs (F = 1.772, p = 0.179) and conflict vehicle AOI (F = 1.236, p > 0.1). The gaze duration in the forward roadway AOI was highest under IFOV1 condition (M = 2.987 s, S.D. = 1.161 s), followed by the conditions of IFOV2 (M = 2.445 s, S.D. = 0.792 s) and IFOV3 (M = 2.156 s, S.D. = 1.039 s) as shown in Fig 4-a. Compared with the IFOV2 and IFOV3 conditions, drivers spent more time to gaze at the forward roadway under IFOV1 condition and consequently they spent less time in observing the right AOI, which might lead to a lower likelihood in detecting the conflict vehicle.

**Table 3:** Descriptive statistical results and ANOVA analysis results for GD in different AOIs.

AOI	Factors		Count	Mean	S.D. ^a^	Min	Max	F-ratio	P-value
Right (s)	IFOV	IFOV1	23	1.581	0.945	0.378	4.118	2.647	0.079
		IFOV2	20	1.917	0.753	0.505	3.286		
		IFOV3	23	2.169	0.994	0.582	4.291		
	Gender	Male	31	1.553	0.745	0.378	3.002	8.437	0.005
		Female	35	2.184	0.985	0.727	4.291		
	Total		66	1.888	0.93	0.378	4.291		
Forward roadway (s)	IFOV	IFOV1	23	2.987	1.161	0.575	4.431	4.130	0.021
		IFOV2	20	2.445	0.792	1.215	3.954		
		IFOV3	23	2.156	1.039	0.371	4.089		
	Gender	Male	31	2.856	0.872	1.471	4.431	6.144	0.016
		Female	35	2.247	1.145	0.371	4.215		
	Total		66	2.533	1.063	0.371	4.431		
Left (s)	IFOV	IFOV1	23	0.173	0.335	0	1.325	0.693	0.504
		IFOV2	20	0.325	0.44	0	1.661		
		IFOV3	23	0.306	0.554	0	2.487		
	Gender	Male	31	0.235	0.291	0	0.905	0.271	0.604
		Female	35	0.293	0.559	0	2.487		
	Total		66	0.266	0.451	0	2.487		
Total (s)	IFOV	IFOV1	23	4.741	0.211	4.206	4.983	1.772	0.179
		IFOV2	20	4.683	0.17	4.271	4.983		
		IFOV3	23	4.631	0.194	4.176	4.983		
	Gender	Male	31	4.644	0.225	4.176	4.983	2.516	0.118
		Female	35	4.721	0.162	4.271	4.983		
	Total		66	4.685	0.196	4.176	4.983		
Conflict vehicle (s)	IFOV	IFOV1	23	1.281	0.677	0.378	2.746	1.236	0.298
		IFOV2	20	1.373	0.786	0.162	3.262		
		IFOV3	23	1.618	0.886	0.582	4.291		
	Gender	Male	31	1.127	0.578	0.259	2.390	9.204	0.004
		Female	35	1.691	0.862	0.162	4.291		
	Total		66	1.426	0.789	0.162	4.291		

^a^ S.D. = standard deviation.

Gender significantly influenced gaze duration in the right AOI (F = 8.437, p = 0.005), forward roadway AOI (F = 6.144, p = 0.016) and conflict vehicle AOI (F = 9.204, p = 0.004), while there was no significant effect on the left AOI (F = 0.271, p > 0.1) and total AOIs (F = 2.516, p = 0.118). Fig 4-b showed that male drivers spent more time in gazing at the forward roadway than female drivers (M = 2.856 s, S.D. = 0.872 s vs. M = 2.247 s, S.D. = 1.145 s), and male drivers had less time to observe the right AOI than female drivers (M = 1.553 s, S.D. = 0.745 s vs. M = 2.184 s, S.D. = 0.985 s). Fig 4-b also showed that female drivers spent longer gaze duration (M = 1.691 s, S.D. = 0.862 s vs. M = 1.127 s, S.D. = 0.578 s) in the conflict vehicle AOI than male drivers during the process of collision avoidance. The longer gaze duration of female drivers in the conflict vehicle AOI and the right AOI implied that female drivers paid more attention to the conflict vehicle or the potential hazardous area than male drivers in terms of longer gaze duration.

The gaze duration was also significantly different between collision group and non-collision group in the right AOI (F = 23.863, p < 0.001), forward roadway AOI (F = 38.099, p < 0.001), total AOIs (F = 7.315, p = 0.009) and conflict vehicle AOI (F = 9.764, p = 0.003). Compared with the collision group, drivers in non-collision group had a longer gaze duration in the right AOI (M = 2.251 s, S.D. = 0.858 s vs. M = 1.252 s, S.D. = 0.683 s), shorter gaze duration in the forward roadway AOI (M = 2.046 s, S.D. = 0.890 s vs. M = 3.386 s, S.D. = 0.768 s), shorter gaze duration in total AOIs (M = 4.638 s, S.D. = 0.196 s vs. M = 4.767 s, S.D. = 0.170 s) and longer gaze duration in the conflict vehicle AOI (M = 1.642 s, S.D. = 0.852 s vs. M = 1.049 s, S.D. = 0.484 s) as shown in Fig 4-c. It shows that drivers would be in a safer situation if they had shorter gaze duration in the forward roadway AOIs but more gaze duration in the right AOI especially on the conflict vehicle during the process of collision avoidance.

### Number of gazes (NG)

**Fig 5:**
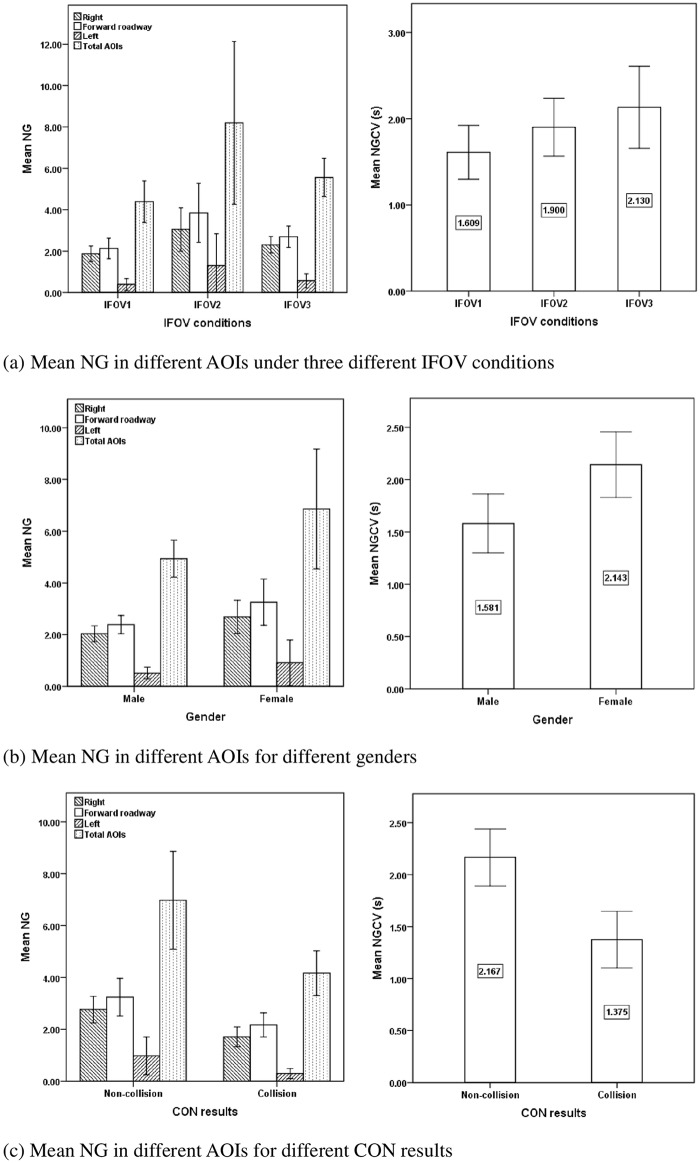
Mean NG in different AOIs for different factors and CON results. (a) Mean NG in different AOIs under three different IFOV conditions. (b) Mean NG in different AOIs for different genders. (c) Mean NG in different AOIs for different CON results.

Table 4 shows the descriptive statistics and ANOVA results for the number of gazes in different AOIs during the process of collision avoidance. The IFOV conditions were found to significantly influence the number of gazes in the right AOI (F = 3.373, p = 0.041) and forward roadway AOI (F = 4.033, p = 0.023), while there was no significant effect on the left AOI (F = 1.129, p > 0.1), total AOIs (F = 2.924, p = 0.061) and conflict vehicle AOI (F = 2.226, p > 0.1). The number of gazes in the right AOI (M = 3.05, S.D. = 2.24), forward roadway (M = 3.85, S.D. = 3.05) AOI were highest under the IFOV2 condition, followed by the conditions of IFOV3 (right AOI: M = 2.30, S.D. = 0.93; forward roadway AOI: M = 2.70, S.D. = 1.18) and IFOV1 (right AOI:M = 1.87, S.D. = 0.87; forward roadway AOI: M = 2.13, S.D. = 1.14) as shown in Fig 5-a. A possible explanation is that compared with the IFOV3 condition, the more restricted field of view in IFOV2 condition caused drivers to scan more times in the right AOI to identify the potential hazardous vehicle; however, the most serious field of view restriction in the IFOV1 condition might prevent the drivers from detecting the conflict vehicle. Therefore, the number of gazes in the right AOI was highest in IFOV2 condition but lowest in IFOV1 condition.

**Table 4:** Descriptive statistical results and ANOVA analysis results for NG in different AOIs.

AOI	Factors		Count	Mean	S.D. ^a^	Min	Max	F-ratio	P-value
Right (s)	IFOV	IFOV1	23	1.87	0.87	1	3	3.373	0.041
		IFOV2	20	3.05	2.24	1	12		
		IFOV3	23	2.30	0.93	1	5		
	Gender	Male	31	2.03	0.84	1	3	3.562	0.064
		Female	35	2.69	1.86	1	12		
	Total		66	2.38	1.5	1	12		
Forward roadway (s)	IFOV	IFOV1	23	2.13	1.14	1	6	4.033	0.023
		IFOV2	20	3.85	3.05	1	16		
		IFOV3	23	2.7	1.18	1	6		
	Gender	Male	31	2.39	0.95	1	4	3.590	0.063
		Female	35	3.26	2.59	1	16		
	Total		66	2.85	2.03	1	16		
Left (s)	IFOV	IFOV1	23	0.39	0.66	0	2	1.129	0.330
		IFOV2	20	1.3	3.28	0	15		
		IFOV3	23	0.57	0.79	0	3		
	Gender	Male	31	0.52	0.63	0	2	0.806	0.373
		Female	35	0.91	2.56	0	15		
	Total		66	0.73	1.91	0	15		
Total (s)	IFOV	IFOV1	23	4.39	2.33	2	11	2.924	0.061
		IFOV2	20	8.2	8.41	3	43		
		IFOV3	23	5.57	2.13	2	11		
	Gender	Male	31	4.94	1.95	2	8	2.631	0.11
		Female	35	6.86	6.75	2	43		
	Total		66	5.95	5.15	2	43		
Conflict vehicle (s)	IFOV	IFOV1	23	1.61	0.72	1	3	2.226	0.117
		IFOV2	20	1.90	0.72	1	3		
		IFOV3	23	2.13	1.10	1	5		
	Gender	Male	31	1.58	0.76	1	4	8.243	0.006
		Female	35	2.14	0.91	1	5		
	Total		66	1.88	0.89	1	5		

^a^ S.D. = standard deviation.

Gender had no significant influence on the number of gazes in different AOIs including the right AOI (F = 3.562, p = 0.064), the forward roadway AOI (F = 3.590, p = 0.063), the left AOI (F = 0.806, p > 0.1), and total AOIs (F = 2.631, p > 0.1), but it significantly influenced the number of gazes in the conflict vehicle AOI (F = 8.243, p = 0.006). Fig 5-b showed that female drivers had more numbers of gazes (M = 2.14, S.D. = 0.91 vs. M = 1.58, S.D. = 0.76) in the conflict vehicle AOI than male drivers during the process of collision avoidance, implying that female drivers paid more attention to the conflict vehicle than male drivers in terms of the number of gazes.

The number of gazes was significantly different between collision group and non-collision group in the right AOI (F = 8.438, p = 0.005), the forward roadway AOI (F = 4.471, p = 0.038), total AOIs (F = 4.815, p = 0.032) and conflict vehicle AOI (F = 14.776, p < 0.001). Compared to the collision group, drivers in the non-collision group had a higher number of gazes in the right AOI (M = 2.76, S.D. = 1.64 vs. M = 1.71, S.D. = 0.91), forward roadway AOI (M = 3.24, S.D. = 2.34 vs. M = 2.17 times, S.D. = 1.09), total AOIs (M = 6.98, S.D. = 6.06 vs. M = 4.17, S.D. = 2.04) and conflict vehicle AOI (M = 2.17, S.D. = 0.88 vs. M = 1.38, S.D. = 0.65), as shown in Fig 5-c. It implied that the drivers who scanned more times when approaching the intersection were less likely to have a collision.

### Average gaze duration (AGD)

**Fig 6:**
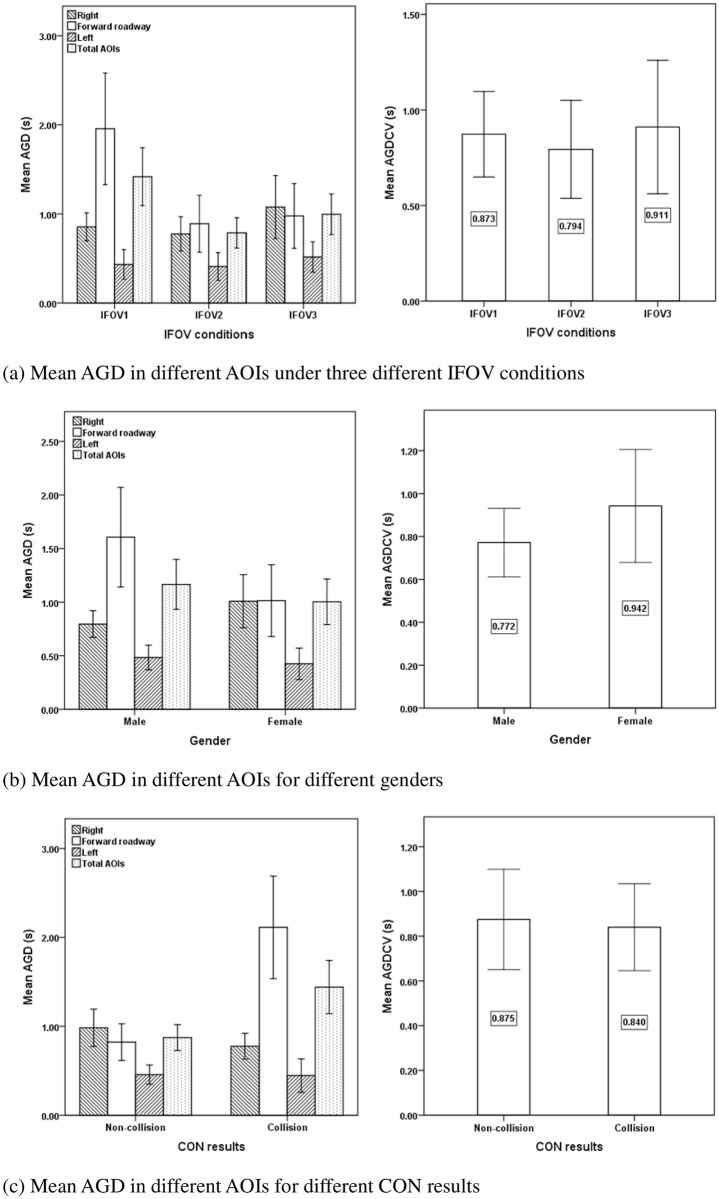
Mean AGD in different AOIs for different factors and CON results. (a) Mean AGD in different AOIs under different IFOV conditions. (b) Mean AGD in different AOIs for different genders. (c) Mean AGD in different AOIs for different CON results.

Table 5 shows the descriptive statistics and ANOVA results for the average gaze duration in different AOIs during the process of collision avoidance. The IFOV conditions significantly influenced the average gaze duration in the forward roadway AOI (F = 7.615, p = 0.001) and total AOIs (F = 6.836, p = 0.002), while there was no significant effect on the right AOI (F = 1.550, p > 0.1), left AOI (F = 0.391, p > 0.1) and conflict vehicle AOI (F = 0.130, p > 0.1). The average gaze duration in the forward roadway AOI (M = 1.956 s, S.D. = 1.450 s) and total AOIs (M = 1.418 s, S.D. = 0.750 s) were highest under the IFOV1 condition, followed by the conditions of IFOV3 (forward roadway: M = 0.977 s, S.D. = 0.840 s; total AOIs: M = 0.996 s, S.D. = 0.528 s) and IFOV2 (forward roadway: M = 0.890 s, S.D. = 0.684 s; total AOIs: M = 0.787 s, S.D. = 0.363 s) as shown in Fig 6-a. It implied that drivers with longer average gaze durations in the IFOV1 condition would have a slower visual search speed in total AOIs; and the drivers would especially have a slower visual search speed in the forward roadway AOI.

**Table 5:** Descriptive statistical results and ANOVA analysis results for AGD in different AOIs.

AOI	Factors		Count	Mean	S.D. ^a^	Min	Max	F-ratio	P-value
Right (s)	IFOV	IFOV1	23	0.855	0.366	0.378	1.761	1.550	0.221
		IFOV2	20	0.776	0.412	0.136	1.622		
		IFOV3	23	1.077	0.819	0.520	4.291		
	Gender	Male	31	0.795	0.341	0.325	1.615	2.156	0.147
		Female	35	1.008	0.723	0.136	4.291		
	Total		66	0.908	0.582	0.136	4.291		
Forward roadway (s)	IFOV	IFOV1	23	1.956	1.450	0.217	4.431	7.615	0.001
		IFOV2	20	0.890	0.684	0.112	3.134		
		IFOV3	23	0.977	0.840	0.186	4.089		
	Gender	Male	31	1.606	1.268	0.490	4.431	5.384	0.024
		Female	35	1.014	0.975	0.112	4.089		
	Total		66	1.292	1.153	0.112	4.431		
Left (s)	IFOV	IFOV1	7	0.433	0.212	0.162	0.727	0.391	0.681
		IFOV2	11	0.410	0.247	0.111	0.929		
		IFOV3	10	0.517	0.260	0.162	0.905		
	Gender	Male	14	0.483	0.210	0.143	0.905	0.135	0.717
		Female	14	0.425	0.271	0.111	0.929		
	Total		28	0.454	0.240	0.111	0.929		
Total (s)	IFOV	IFOV1	23	1.418	0.750	0.415	2.492	6.836	0.002
		IFOV2	20	0.787	0.363	0.116	1.650		
		IFOV3	23	0.996	0.528	0.417	2.492		
	Gender	Male	31	1.165	0.636	0.551	2.492	1.264	0.265
		Female	35	1.004	0.618	0.116	2.492		
	Total		66	1.080	0.627	0.116	2.492		
Conflict vehicle (s)	IFOV	IFOV1	23	0.873	0.517	0.378	2.530	0.130	0.878
		IFOV2	20	0.794	0.549	0.081	2.005		
		IFOV3	23	0.911	0.880	0.404	4.291		
	Gender	Male	31	0.772	0.436	0.259	2.005	0.969	0.329
		Female	35	0.942	0.766	0.081	4.291		
	Total		66	0.862	0.634	0.081	4.291		

^a^ S.D. = standard deviation.

Gender significantly influenced the average gaze duration in the forward roadway AOI (F = 5.384, p = 0.024), while the effects on the right AOI (F = 2.156, p > 0.1), left AOI (F = 0.135, p > 0.1), total AOIs (F = 1.264, p > 0.1) and conflict vehicle AOI (F = 0.969, p > 0.1) were not significant. Fig 6-b showed that female drivers spent a shorter average gaze duration per observation time in the forward roadway AOI than male drivers (M = 1.014 s, S.D. = 0.975 s vs. M = 1.606 s, S.D. = 1.268 s), which implied that female drivers would have a faster visual search speed on the forward roadway than male drivers.

The average gaze duration was also significantly different between collision group and non-collision group in the forward AOI (F = 26.722, p < 0.001) and total AOIs (F = 15.283, p < 0.001). Compared with the collision group, drivers in non-collision group had a shorter average gaze duration in the forward roadway AOI (M = 0.822 s, S.D. = 0.663 s vs. M = 2.113 s, S.D. = 1.366 s) and in total AOIs (M = 0.873 s, S.D. = 0.469 s vs. M = 1.441 s, S.D. = 0.710 s), as shown in Fig 6-c. It indicated that the collision risk of drivers would be reduced if they had faster visual search speed in terms of shorter average gaze duration in the total AOIs, especially in the forward roadway AOI during the process of collision avoidance.

## Discussion

### Relationship between eye movements and traffic safety

Drivers’ visual information acquisition and attention was realized by drivers’ scanning performance. A failure to scan the information of the roadway has been identified as a major causation of traffic crashes [31, 32]. Inefficient perceptual processing in the driving situation may be partly due to the lack of abilities of detecting objects in the traffic environment and general information acquisition and attention [33]. In this study, the experiment results showed that the drivers who scanned more times at the intersections attempted to collect more critical visual information about the presence of traffic in crossing lanes, and thus had a lower collision rate. It implies that the better scanning performance of drivers would enhance their crash avoidance abilities. The finding strengthens the previous evidence that drivers who had more scan times were more likely to detect and monitor potential hazard location [34].

In fact, a variety of sources have established a link between drivers’ scan patterns and attention. Underwood reported that drivers’ attention which was focused on the road straight ahead was detrimental for scanning and judging the neighboring traffic situation [34], which may increase the possibility of a driver being involved in a collision when encountering an abruptly appearing hazard. In this study, the experimental results indicated that when approaching the intersections, the drivers who focused more on the forward roadway and paid less attention to the conflict vehicle emerging area were more likely to have collisions. The finding is consistent with the previous conclusion that less allocations of visual attention to the potential hazardous areas impaired drivers’ observation of critical visual information and caused higher collision rates [35, 36]. It was reported that 78% of crashes contained at least one type of inattention [37], and inattention was also considered as one of the most fatal causes of road traffic accidents [15]. Additionally, this simulation study showed that drivers who had a faster visual search speed in terms of a shorter average gaze duration in the total AOIs (especially the forward roadway) were more likely to successfully avoid collisions. This finding is consistent with the results of visual search efficiency for driving safety in previous studies [38, 39].

Effect of IFOV conditions on eye movements Previous studies have confirmed that drivers tend to perform safety-related adaptations to deal with limited visual field, such as reducing speed to compensate [40, 41]. This study further explored the effects of IFOV conditions on drivers’ eye movements and visual attention on the particular AOI and the relationship between traffic safety and drivers’ eye movement.

The limited IFOV would impede the drivers’ abilities to obtain visual information correctly and on time. It was found that as IFOV increased, the drivers paid less attention to gaze at the forward roadway, indicating that the drivers made more efforts to observe the critical potential risk information around the intersections. A longer gaze duration meant that drivers could gain the most important visual information about target AOIs [42]. The results in this study prove that more critical visual information could be obtained at intersections through improving IFOV. Similar to the findings in UFOV research, the larger UFOV was effective in improving visual attention skills [43] and driver’s ability of visual information acquisition [44].

Moreover, there was an interesting finding that drivers scanned more frequently on the right side of intersection under the IFOV2 condition than the other two IFOV conditions. One reason might be that the drivers under the IFOV2 condition could not observe the conflict vehicle as clearly as the IFOV3 condition, but they could still observe the conflict vehicle earlier than the IFOV1 condition. Previous studies had shown that more scan times for a search task occurred when the area of interest was difficult to understand and the visual environment was more complex [45, 46]. Because the horizontal visibility in the IFOV2 condition was lower than IFOV3, drivers need to more frequently scan around the intersection for the critical visual information in the IFOV2 condition. Furthermore, although the most restrictive IFOV1 condition satisfied the current intersection design standards in AASHTO [5], it had the least number of gazes and the longest gaze duration, in which drivers had the slowest visual search speed in capturing intersection information and thus led to the highest collision rate. From the perspective of drivers’ visual performance during the crash avoidance, the findings support the conclusion that the larger IFOV should be encouraged in practical applications by removing any sight obstructions or broadening the non-signalized intersections to increase drivers’ horizontal visibility and reduce collision [6].

### Effect of gender on eye movements

As one of typical driver characteristics, gender is associated with driving performance and crash involvement [47–49]. A previous driving simulator study showed that gender is a significant factor influencing gap acceptance behavior at the intersection and male drivers tend to aggressively accept smaller gaps to merge into traffic than female drivers [50]. In this study, it was found that male drivers had a higher COR than female drivers, which was consistent with previous studies [33, 51]. The possible reason is that female drivers had less risky driving behaviors than male drivers [52, 53] and male drivers tend to be excessively optimistic on their driving skills and usually behave less cautiously than female drivers [54].

In this experiment, the visual search performance during the process of collision avoidance demonstrated that compared with male drivers, female drivers had better abilities to obtain visual information correctly and timely, as indicated by more visual attention and higher visual search frequency at intersections. Specifically, female drivers had more numbers of gazes and longer gaze duration on the conflict vehicle and the right side of intersection than male drivers, indicating that female drivers had a higher visual search frequency for the critical visual information than males in the emergent situation. Thus, it can be inferred from the differences in visual search patterns that female drivers are more careful about potential risk than male drivers.

### Limitation of this study

In this paper, the simulation results reflect drivers’ eye scanning activities and driving performance during emergent collision avoidance. However, it should be mentioned that for a simulator experiment, there are still some general limitations and validation considerations. After all, a simulator test is not driving in the real world and the participants knew that their driving errors would not affect their safety. However, from an ethical point, researchers could not put the participants in a real dangerous driving environment to examine their collision avoidance performance. Even though there might be differences between results observed in a field test and simulated driving, numerous studies have proved that driving simulators provide an adequate representation of the real world and it is a proper tool to be used in driving performance studies [55–57]. In addition, since eye movements are assumed to represent a shift in visual attention, a further study is suggested to develop matrix models for interpreting and evaluating eye-movements to predict drivers’ intent and actions interactively. Thus, future vehicles technology development could consider equipping the vehicle with eye tracking devices for detecting drivers’ unsafe distractions through monitoring drivers’ fixation patterns.

## Conclusions

In summary, the experiment illustrated the effects of IFOV and gender on drivers’ eye movements during a collision avoidance process when approaching a non-signalized intersection. The findings identified the relationships between drivers’ eye movements, IFOV and crash risk at non-signalized intersection. In this study, better scanning performance was found to have positive effect on driving safety. During the process of emergent collision avoidance, drivers who scanned the intersection surroundings more frequently, paid more visual attention to the potential conflict and had a faster visual search speed were less likely to collide with the conflict vehicle. As the IFOV conditions improved, more critical visual information could be effectively captured by drivers, and thus they could brake earlier to avoid collision with the conflict vehicle, which resulted in a lower crash risk at intersections. Moreover, female drivers had a higher visual search frequency and paid more visual attention to the potential hazard, and also had a faster visual search speed in scanning the critical visual information than male drivers, which explained the finding that female drivers had a lower crash rate than male drivers.

Additionally, compared with non-control intersections, the requirements of sight distance design, traffic rules, drivers’ visual search patterns, and intersection environments are different in yield/stop controlled and signalized intersections. The findings of this study may not be able to directly transfer into the other types of intersections. However, the research method can be applied in more scenarios to comprehensively establish the relationship between IFOV, drivers’ eye movement performance, driving behavior, and crash risk at intersections. Finally, the study results highlight that it is important to investigate how drivers’ eye movements vary as a function of IFOV conditions and suggest that it should be necessary to reconsider the adequacy of the current minimum intersection sight distance design standards.
